# RNA-Seq analysis of differentially expressed genes relevant to innate and adaptive immunity in cecropin P1 transgenic rainbow trout (*Oncorhynchus mykiss*)

**DOI:** 10.1186/s12864-018-5141-8

**Published:** 2018-10-19

**Authors:** Yueh-Chiang Han, Chun-Mean Lin, Thomas T. Chen

**Affiliations:** Department of Molecular and Cell Biology, University of Connecticut, Storrs, CT 06269 USA

**Keywords:** *Oncorhynchus mykiss*, Disease resistant transgenic fish, Antimicrobial peptide, Cecropin P1, RNA-Seq

## Abstract

**Background:**

In the past years, our laboratory successfully generated transgenic rainbow trout bearing cecropin P1 transgene. These fish exhibited resistant characteristic to infection by *Aeromonas salmonicida,* Infectious Hematopoietic Necrosis Virus (IHNV) and *Ceratomyxa shasta* (a parasitic pathogen). Previously, treating rainbow trout macrophage cells (RTS-11) with cecropin B, pleurocidin and CF17, respectively, resulted in elevated expression of two pro-inflammatory genes, e.g. cyclooxygenase-2 (*cox-2*) and interleukin-1β (*il-1β*). In addition, a profiling of global gene expression by 44 k salmonid microarray analysis was conducted, and the results showed that immune relevant processes have been perturbed in cecopin P1 transgenic rainbow trout. Therefore, we hypothesized that cecropin P1 may not only eliminate pathogens directly, but also modulate the host immune systems, leading to increased resistance against pathogen infections. To confirm this hypothesis, we performed de novo mRNA deep sequencing (RNA-Seq) to analyze the transcriptomic expression profiles in three immune competent tissues of cecropin P1 transgenic rainbow trout.

**Results:**

De novo sequencing of mRNA of the rainbow trout spleen, liver and kidney tissues were conducted by second-generation Illumina system, followed by Trinity assembly. Tissue specific unigenes were obtained, and annotated according to the Gene Ontology (GO) and the Nucleotide Basic Local Alignment Search Tool (BLAST). Over 2000 differentially expressed genes (DEGs) were determined by normalized ratio of Reads Per Kilobase of transcript per million mapped reads (RPKM) among the transgenic and non-transgenic fish in a tissue specific manner, and there were 82 DEGs in common among the three tissues. In addition, the enrichment analysis according to Gene Ontology Biological Process (GO:BP), and Kyoto Encyclopedia of Genes and Genomes (KEGG) based pathway analysis associated with innate/adaptive immunity of fish were also performed to illustrate the altered immune-related functions in each tissue.

**Conclusions:**

According to the RNA-Seq data, the correlations between alteration of gene expression profiles and the functional perturbations of the host immune processes were revealed. In comparison with the results of cDNA microarray analysis conducted by Lo et al., the overall results supported our hypothesis that the gene product of cecropin P1 transgene may not only directly eliminate pathogens, but also modulate the host immune system. Results of this study present valuable genetic information for *Oncorhynchus mykiss*, and will benefit future studies on the immunology of this fish species.

**Electronic supplementary material:**

The online version of this article (10.1186/s12864-018-5141-8) contains supplementary material, which is available to authorized users.

## Background

Outbreak of diseases caused by pathogen infection is one of the most serious bottlenecks in the aquaculture industry worldwide. Traditional strategies including vaccination, treatment of diseased fish with antibiotics, and artificial selection of disease resistant fish strains by traditional approach have been used to control fish diseases. However, deficiencies, such as high economic costs of vaccination, selection of antibiotic-resistant pathogens, lacking effective cure of viral infection, and low degree of overall protection of fish population associated with these traditional strategies have limited their feasibilities. Therefore, effective means of controlling fish diseases in the aquaculture industry are awaiting to be developed, and transgenic fish technology may provide a solution to this problem. Mediated via the transgenic fish technology, directly modifying the unwanted genetic traits that cause the vulnerability of fish to infection by pathogens, or introducing specific genes that may confer resistance to pathogen infection into the fish genome might achieve the purpose of protecting fish from invasion by pathogens (see review by Chen et al.*,* 2014) [1].

Cecropin B, which was first identified in cecropia moth, *Hyalophora cecropia*, is one of the antimicrobial peptide (AMP) family member proteins playing an essential role in the innate immunity of insects [2]. According to Shai [3], the zwitterionic AMPs, 31 to 37 amino acid residues in length and α-helical cationic amphipathic peptides, can be integrated into the cellular membrane of pathogens and resulted in the formation of pores on the cellular membrane leading to ultimate lysis of pathogen cells [4]. Cecropin P1, identified from nematode inhabiting in the porcine small intestine, was found to be more potent against Gram-negative bacteria than Gram-positive bacteria [5], and was thus chosen as transgenic target gene in our laboratory for production of transgenic rainbow trout.

In the past years, transgenic medaka [6] and rainbow trout [7] that harbor cecropin B or cecropin P1 transgene were generated by microinjecting the transgene into fertilized eggs or by electroporating the transgene into sperm. Through repeated challenge studies, families of heterozygous cecropin P1 transgenic rainbow trout in the second and third generations were shown to be resistant to infection by *Aeromonas salmonicida*, infectious hematopoietic necrosis virus (IHNV) and *Ceratomyxa shasta* (a common parasite infecting rainbow trout) [7, 8]. In addition, Chiou et al. demonstrated that in vitro treatment of rainbow trout RTS11 cells (a trout macrophage cell line) with cecropin B and its synthetic analogue (CF-17) led to up-regulation of interleukin-1β (*il-1β*), which is a pro-inflammatory cytokine, and cyclooxygenase (*cox-2*), an enzyme essential for the inflammatory modulation [9]. Although AMPs can eliminate pathogens directly, Chiou et al. [10] and Lai [11] further suggested that AMPs might also initiate an immune-modulatory process in the host immune system. To support this perception, a preliminary study on the analysis of gene expression profiles of immune related genes in two families of cecropin P1 transgenic rainbow trout was conducted by Lo et al. [12] in our laboratory via cDNA microarray analysis on a 44 k custom made salmonid chip [13]; many tissue-specific differentially expressed genes (DEGs), namely in the spleen, liver, and kidney, were determined. Organ specific functional perturbations of the host innate/adaptive immune pathways were identified including phagocytosis, lysosomal processing, complement activation, antigen presenting, and leukocyte migration. Furthermore, disturbance of biological processes, which may contribute indirectly to host immunity, were also determined such as lipid metabolic process, cellular focal adhesion, and extracellular matrix (ECM)-organization [12]. By combining these facts, we hypothesized that transgenic cecropin P1 may not only eliminate pathogens directly, but also modulate the host’s innate and adaptive immunity. However, cDNA microarray conducted by Lo et al. has several limitations, e.g. (1) hybridization based florescence detection limits the measurement accuracy of the expression levels, especially the transcripts existing in very high or low copy numbers [14]; (2) only 44 k genes were analyzed and didn’t provide a global view of the total transcriptome; (3) the gene chip was customized according to *Salmo salar* genome and considered not thoroughly reflecting the trout genomic information. To overcome these limitations, and to extend the study for more solid evidence, a cross-platform confirmation is highly desirable.

The recent advances of the next generation sequencing technology (NGS), i.e., deep RNA sequencing (RNA-Seq), has exerted tremendous impact on studies on “de novo” construction of the transcriptome without a reference genome, evaluation of nucleotide variations, and evaluation of methylation patterns of genes etc. [15–17]. This technology has advantages over the cDNA microarray analysis in the following aspects: firstly, high levels of data reproducibility leading to reduction of technical replications for the experiments; secondly, allowing easy identification and quantification of the expression of isoforms and unknown transcripts; thirdly, increasing the popularity of high throughput sequencing technologies resulted in a significant reduction of the cost the RNA-Seq experiments. To confirm the mentioned hypothesis in the current study, de novo mRNA deep sequencing by the Illumina second generation system was carried out in three different immune relevant tissues (namely the spleen, liver, and kidney) of cecropin P1 transgenic rainbow trout, and followed by Trinity assembly of the data [18]. By sorting of Reads Per Kilobase of transcript per million mapped reads (RPKM), the DEGs were determined. From the GeneCodis enrichment analysis [19, 20], we discovered functional alterations of biological processes with identifiers of Gene Ontology: Biological Process (GO:BP) and Kyoto Encyclopedia of Genes and Genomes (KEGG) databases. Finally, by establishing KEGG-based pathways analyses, we further revealed that immune relevant processes in the spleen and kidney, and energy metabolism relevant processes in the liver were significantly perturbed. These results strongly support our hypothesis, and will benefit future studies on the genetics of fish immunology. Furthermore, these perturbed DEGs may also serve as biological markers for artificial selection and breeding of disease resistant aquaculture important fish species.

## Results

### De novo sequencing, reads assembly, and annotation of reference database from non-transgenic fish

Pools of RNA samples from three tissues (i.e., the spleen, liver, and kidney) of non-transgenic rainbow trout were used to generate the reference genome library database. By removing reads of adaptor, reads of unknown nucleotides greater than 5% and reads with low quality bases (base quality ≦ 10), 103,048,352 paired-end clean reads from total nucleotides of 9,274,351,680 were obtained. After de novo assembly by the Trinity program [18], 257,490 contigs with the average length of 285 bp were collected, and a total of 141,850 unigenes with the average length of 523 bp were determined (Table 1). Subsequently, the unigenes were annotated by BLAST-p according to six databases, namely nucleotide collection NR/NT, Swiss-Prot, KEGG, GO, and Clusters of Orthologous (COG). The annotated unigenes were than used as reference genome library for further transgenic transcriptomic sequence mapping and assembly.

**Table 1:** Summary of non-transgenic rainbow trout transcriptome

Reads	Numbers
Total Raw Reads	115,871,414
Total Clean Reads	103,048,352
Total Clean Nucleotides (nt)	9,274,351,680
Total Contigs	257,490
Total Length of Contig	73,387,555
Avg. Length of Contig (nt)	285
Total Unigenes	141,850
Avg. Length of Unigene (nt)	523
Total Length of Unigene	74,181,833

### Sequencing and assembly of cecropin P1 transgenic rainbow trout

Messenger RNA isolated from three tissues (the spleen, liver, and kidney) of cecropin P1 transgenic rainbow trout were individually sequenced by Illumina HiSeq 2000. By eliminating reads of adaptors, reads of unknown nucleotides larger than 10% and reads with low quality bases (base quality ≦ 5) greater than 50%, clean reads were obtained in a tissues specific manner. Afterwards, the clean reads were mapped to reference database generated from non-transgenic control by the aligning program of short oligonucleotide analysis package: SOAPaligner/SOAP2 [21] with no more than two mismatches allowed to generate tissues specific unigenes. A sum of 131,671 unigenes was determined, and the overall good qualities of reads and aligning results were summarized in the Additional file 1.

### Determination of differential expression profiles

**Fig. 1:**
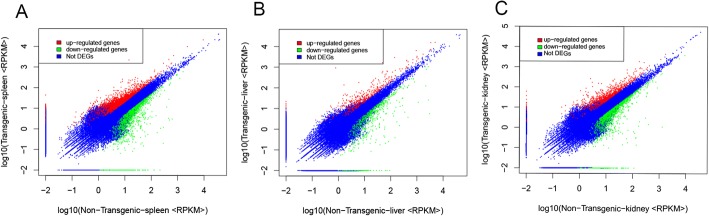
Differential gene expression patterns identified in transgenic and non-transgenic fish. The gene expression levels were represented by plotting base 10 logarithm of RPKM values of non-transgenic (x-axis) against transgenic (y-axis). Red = up-regulated genes, green = down-regulated genes and blue = no change among transgenic and non-transgenics. **a** Spleen; **b** Liver; **c** Kidney. Thresholds set for all plots: FDR < 0.001 and |log_2_ (RPKM ratio)| > 1

**Fig. 2:**
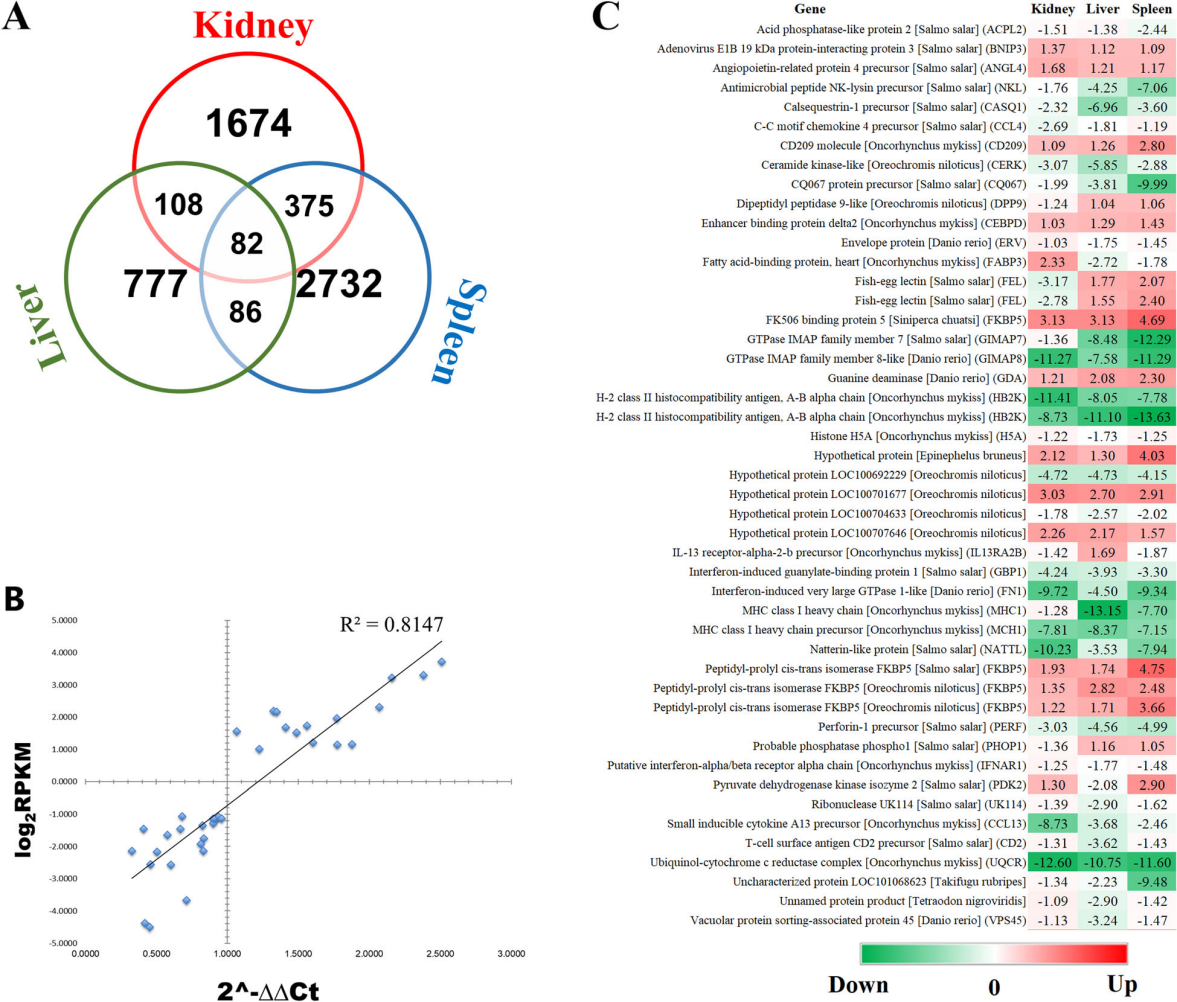
A global view of RNA-Seq data analysis among three immune competent tissues of cecropin P1 transgenic rainbow trout. **a** Venn chart shows numbers of differentially expressed genes (DEGs) determined in each tissue. **b** Confirmation of RNA-Seq expression level via real time RT-qPCR assays (*n* = 36; linear regression with R^2^ = 0.81). **c** Selective list of genes with annotations from the 82 DEGs in common among three tissues. The heat-map denotes the relative folds of expression in each gene, the degree of expression ratio is represented by |log_2_ (RPKM ratio)| > 1

By calculating the ratio of RPKM of unigenes [22] among transgenic and non-transgenic fish, the differentially expressed gene profiles were determined with a threshold of two folds been set. The genes with RPKM ratio greater than two folds (transgenic/non-transgenic > 2 or < 0.5) were considered as significantly up- or down-regulated, respectively, and were defined as differentially expressed genes (DEGs). As shown in Fig. 1, there were 1873/1402 genes in the spleen, 431/622 genes in the liver, and 916/1323 genes in the kidney were determined as significantly up−/down-regulated. After evaluating RPKM ratio in base two logarithm and eliminating redundancy by probes annotated with identical geneID, a total of 3275, 2239 and 1053 DEGs were identified in the spleen, kidney and liver, respectively. Additionally, by sorting RPKM ratio, a sum of 82 DEGs are in common among three tissues, 375 DEGs between the spleen and kidney, 86 DEGs between the spleen and liver, and 108 DEGs between the kidney and liver were identified (Fig. 2a). Moreover, these 82 DEGs belong to immune related biological functions, showing consistent or inverse expression patterns among the three tissues (Fig. 2c), where color gradient denoting relative fold changes of RPKM ratio. To confirm the gene expression level obtained by RNA-Seq, selected genes covering a wide range of expression ratios were determined by real-time quantitative reverse transcription poly chain reaction (RT-qPCR) analysis. By comparing the two independent platforms, a general agreement with an acceptable degree of linear correlation (R^2^ = 0.81, Fig. 2b) was observed, which indicating the expression dataset was solid.

### Enrichment analysis of biology terms of GO and KEGG pathway by GeneCodis

**Fig. 3:**
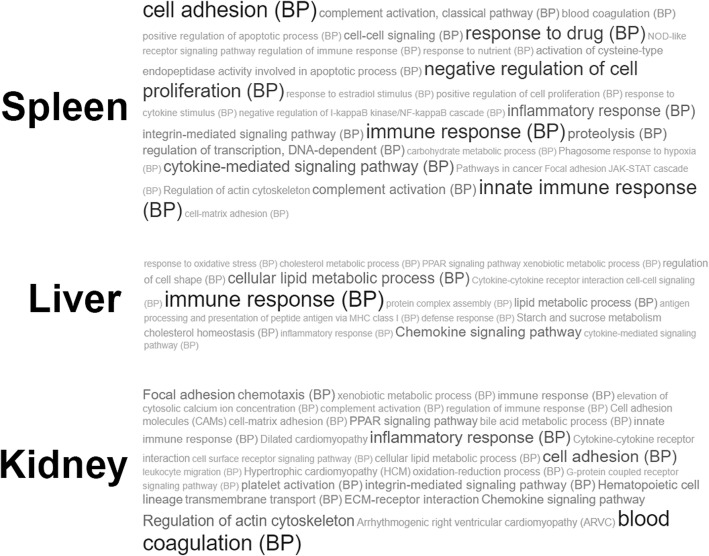
Graphic view of Tag cloud of biological terms from modular enrichment analysis by GeneCodis. The most significant 35 terms acquired from GO: Biological Process (BP) and KEGG database were given for each tissue. The font size of tags indicates the degree of DEGs been enriched for each biological process

To uncover the thematic association of altered gene expression patterns in cecropin P1 transgenic rainbow trout, enrichment analysis by GeneCodis was conducted. GeneCodis was developed as an over-representation analysis (ORA) approach [19, 20] and could be performed in either singular or modular enriching biological terms via different databases. The DEGs of each tissue and the total genes from reference genome were inputted to inquire GO biological process, GOSlim process and KEGG pathways. In Fig. 3, we reported the resulting tag clouds of modular GeneCodis enrichment analyses. The modular analyses, tissue specifically, indicated the intersections of sets of terms of biological processes among all and each of the three different annotation databases. Therefore, it revealed the most conspicuous terms with distinct profiles of enrichment. Afterwards, the statistical significance of terms in each tissue were ranked by hypergeometric and chi-square methods to discover the highly perturbed biological processes in each tissue studied. As shown in Table 2, the top 5 most perturbed biological functions in the spleen of the transgenic fish are cell adhesion, negative regulation of cell proliferation, innate and total immune response, and cytokine-mediated signaling pathways. In the kidney, functional biological processes found greatly altered include peroxisome proliferator-activated receptor (PPAR) signaling, cell adhesion, hematopoietic cell lineage, regulation of immune response, and inflammatory response. In the liver, fewer of the biological terms were enriched such as lipid metabolic process, immune response, and chemokine signaling pathways. As expected, energy efficiency correlated processes were also impacted in the liver of transgenic fish including PPAR signaling, starch and sucrose metabolism, and cholesterol metabolic and homeostasis. To discover a better broad-spectrum of tissue-specific direct and indirect effects on immune relevant DEGs, singular enrichment results were also analyzed (Additional file 2) since singular enrichment analyses enlighten the union of all sets of biological terms in each annotation database. The ECM-receptor interaction and hematopoietic cell lineage were altered in the kidney (Additional file 2, KEGG terms). Finally, starch and sucrose metabolism, chemokine signaling, and cytokine-cytokine receptor interaction were influenced in the liver as well (KEGG terms in the Additional file 2).

**Table 2:** Top 5 GO and KEGG terms of each transgenic tissue via modulatory enrichment with GeneCodis

Biological terms	Genes (I/R)^1^	*P*-value^2^	Identifier
Spleen			
Innate immune response (BP)	7/12	2.68E-04	GO: 0045087
Immune response (BP)	19/86	2.87E-04	GO: 0006955
Cell adhesion (BP)	29/198	1.98E-03	GO: 0007155
Negative regulation of cell proliferation (BP)	20/128	7.18E-03	GO: 0008285
Cytokine-mediated signaling pathway (BP)	13/41	5.83E-04	GO: 0019221
Kidney			
Inflammatory response (BP)	6/8	1.55E-05	GO: 0006954
Regulation of immune response (BP)	8/21	8.65E-05	GO: 0050776
Cell adhesion (BP)	6/23	1.97E-03	GO: 0007155
PPAR signaling pathway	7/23	5.63E-04	KEGG: 03320
Hematopoietic cell lineage	5/10	5.99E-04	KEGG: 04640
Liver			
Cellular lipid metabolic process (BP)	3/11	5.38E-03	GO: 0044255
PPAR signaling pathway			KEGG: 03320
Immune response (BP)	7/86	6.06E-03	GO: 0006955
Chemokine signaling pathway	3/7	3.00E-03	KEGG: 04062
Cholesterol homeostasis (BP)	4/20	3.98E-03	GO: 0042632
Starch and sucrose metabolism	4/25	4.86E-03	KEGG: 00500

^1^I/R stand for numbers of annotated genes in the input list divided by numbers of annotated genes in the reference list; ^2^The *P*-values were calculated by hypergeometric analysis and adjusted by false detection rate (FDR) for multiple corrections

### Analysis of functional perturbed immune relevant pathways

The alteration of biological processes in the spleen, liver and kidney of the transgenic rainbow trout were studied by GeneCodis enrichment analysis. To characterize genes contributing to immune relevant pathways that were affected by cecropin P1 transgene product, and to determine the signaling pathways that has been impacted, the pre-defined unigenes and DEGs from three tissues were subjected to analysis against KEGG pathways in order to assign their functions within the tissue-specific biological processes. By inputting a sum of 37,632 unigenes in all three tissues (the spleen, liver and kidney), 1286 DEGs in the spleen, 465 DEGs in the liver, and 936 DEGs in the kidney into the KEGG pathway database, 239, 212 and 235 KEGG pathways were mapped in the spleen, liver and kidney, respectively. By sorting the ratios among DEGs and all unigenes with pathway annotation (*p*-value < 0.05), followed by combining to the tissue-specific functions, the significantly perturbed immune relevant pathways were summarized in Table 3. Via ranking the ratio in the order of statistical significance (Table 3, I/R ratio), it reveals that out of 735 total annotated unigenes, a sum of 51 DEGs associate to leukocyte trans-endothelial migration, 45/562 to cytokine interaction, 32/493 to Janus kinase (JAK) to signal transducer and activator of transcription (STAT) signaling, and 40/913 to chemokine signaling were impacted in the transgenic spleen. Similarly, several DEGs in the pathways of the complement & coagulation cascade, the Toll-like receptor (TLR), the antigen processing & presentation and Fc ε RI signaling & Fc γ RI-mediated phagocytosis were greatly altered in the spleen. In the kidney, DEGs in the leukocyte trans-endothelial migration and hematopoietic cell lineage pathways were impacted (Table 3). By analyzing liver-associated pathways, DEGs in the pathways of phagosomal activity, fatty acid biosynthesis, and complement & coagulation cascade were significantly perturbed (Table 3). Interestingly, highly disturbed PPAR signaling pathways were discovered in both liver and kidney, and this result may indicate a major alteration in regulating energy efficiency occurred in transgenic rainbow trout.

**Table 3:** Summary of tissue specific pathway analyses of cecropin P1 transgenic rainbow trout via KEGG database

Pathways	DEGs (I/R)^a^	*P*-Values	KEGG ID^b^
Spleen			
Chemokine and Cytokine JAK-STAT	45/562	1.52E-07	KO04060
	40/913	6.69E-03	KO04062
	32/493	4.63E-04	KO04630
Complement and Coagulation cascade	26/325	6.16E-05	KO04610
Toll-like receptor	29/435	5.47E-05	KO04620
Antigen processing and presentation	32/290	6.95E-09	KO04612
Leukocyte trans-epithelial migration	51/735	1.79E-06	KO04670
Fc ε RI signaling and γ R-mediated phagocytosis	29/458	1.22E-03	KO04664
	44/809	1.86E-03	KO04666
Liver			
PPAR signaling	22/325	1.62E-10	KO03320
Phagosome	31/806	3.43E-08	KO04145
Complement and Coagulation cascade	27/325	9.74E-15	KO04610
Fatty acid biosynthesis	6/33	2.88E-06	KO00061
Kidney			
PPAR signaling	39/325	6.13E-16	KO03320
Hematopoietic cell lineage	40/445	3.65E-12	KO04640
Leukocyte trans-epithelial migration	28/735	1.82E-02	KO04670

^a^I/R stands for mapped DEGs in each tissue divided by total annotated genes in the contributing KEGG pathways. ^b^KEGG ID indicates the acknowledgements to the corresponding pathways were modified and sprayed expression data from the original sources

## Discussions

Positively charged and amphipathic AMPs, e.g. cecropin P1, have been characterized for their activities of cytotoxic elimination of bacteria and viruses as their primary function in host innate immunity [23]. As reviewed by Hilchie et al. [24], AMPs were also found to have multifaceted immunomodulatory effects in a variety of different hosts. The immunomodulatory activities of these AMPs were found to exert differential expression of cytokines and chemokines, differentiation of leukocytes, and elevation of damage repairing and so on in animal models [11, 24, 25]. Recently, transgenic rainbow trout bearing cecropin P1 transgene was produced in our laboratory, and the elevation of resistance to bacterial, viral and parasitic infections were also demonstrated through repeated challenge studies [7]. Therefore, it is conceivable to use these transgenic fish as experimental animals to address the question whether cecropin P1 transgene product can modulate host immune system or other genetic traits that may lead to increasing resistant to microbial infections in the host. In a preliminary cDNA microarray study conducted Lo et al. [12], they had unveiled that several functional alterations on the expression of immune related genes have taken place in the spleen, liver, and kidney of the transgenic fish. However, due to the inherited disadvantages associated with the cDNA microarray study, as mentioned in the Introduction, a confirmation study via a second experimental approach is required in order to provide a more solid evidence to support our hypothesis, and thus RNA-Seq analysis was adopted in this study [14].

### Spleen: The major innate/adaptive immune relevant organ

**Fig. 4:**
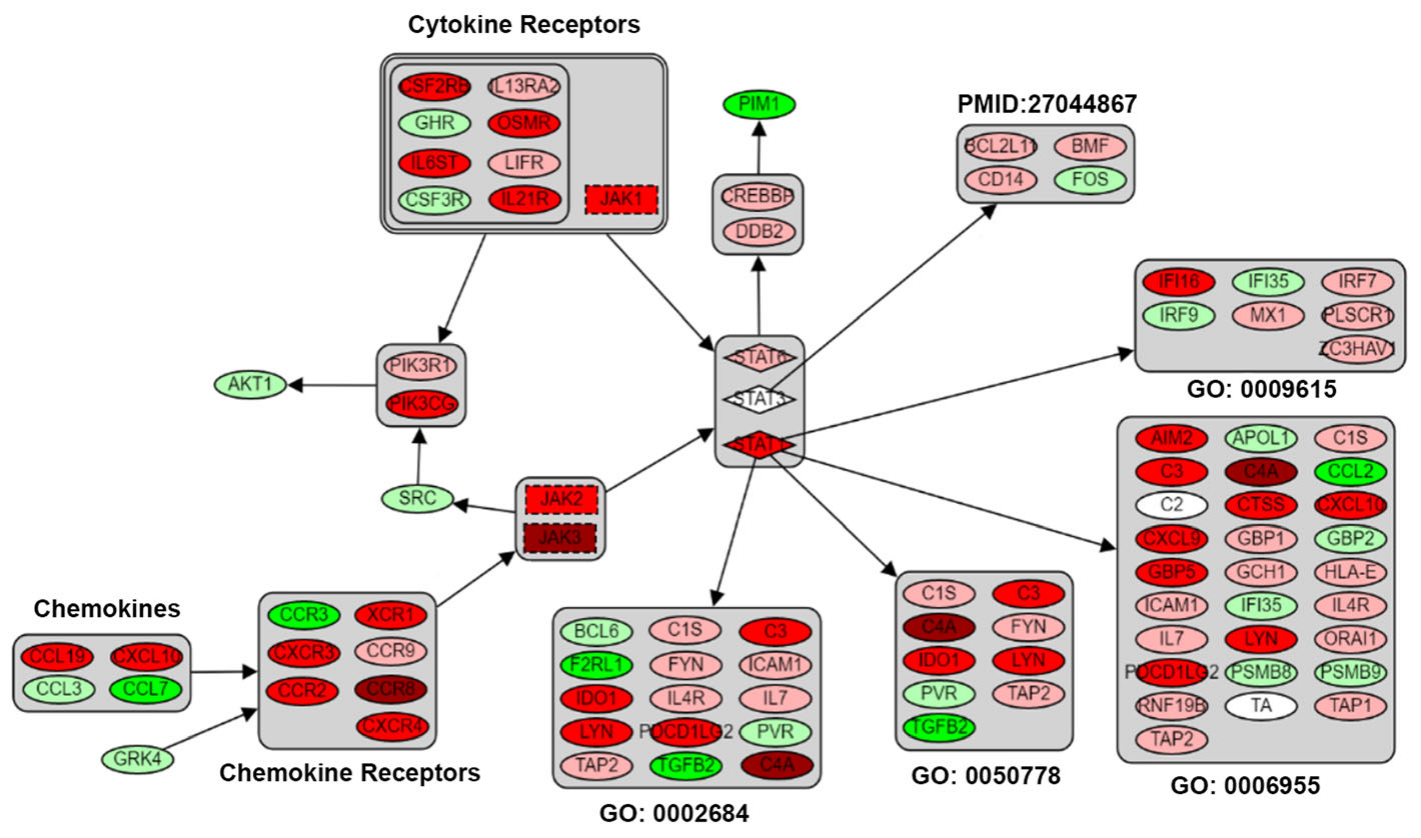
Pathway analysis of cytokine/chemokine driven JAK-STAT pathway in transgenic spleen. The RPKM ratio of DEGs from spleen was sprayed to custom-made cytokine and chemokine mediated JAK-STAT pathway. The red and green heat-map denotes the up- or down-regulation respectively, and the numbers indicate folds change of each DEG. Eclipses refer to regular gene; dash rectangular boxes stand for kinases; diamonds indicate to transcription factors; single bundle refer to set AND set; double bundle refer to AND including sets plus subset. The pathway was customized by combination of KO04060 (cytokine-cytokine receptor interaction), KO04062 (chemokine signaling pathway) and KO04630 (JAK-STAT signaling pathway) from KEGG database. The down-stream STATs target genes were inquired from corresponding GO database as well as research conducted by Sakamoto et al. [31]

The major functions of the spleen in fish had been well reviewed by scientists [26–30]. Very much alike to other higher vertebrates, the spleen of teleost fish is merged in a complex of splenic ellipsoids, melanomacrophages centers (MMCs) and lymphoid tissue [26], this complex filters and traps blood cells, and processes antigens [27]. Other functions of fish spleen are also known to include immune memories mediated by presenting lymphocytic antigen [28], phagocytosis of macrophages stimulated by complement receptors to C3 signaling [29], and naïve CD4^+^ T cells differentiating into T helper cells induced by major histocompatibility complex (MHC) [30]. Since spleen plays critical roles in fish immunity, we believe that one of the major functional perturbations in cecropin P1 transgenic rainbow trout may occur in the spleen. To address this hypothesis, immune relevant pathways were generated by a custom made visualization tool from Shin et al. [31], and mapped with RPKM ratios of pre-defined transgenic spleen DEGs. As excepted, significant alterations in the cytokine/chemokine mediated JAK-STAT signaling pathway (Fig. 4) was observed in the spleen of transgenic rainbow trout. In addition, the heat-map analysis revealed a significant enhancement of cytokine and chemokine signaling via ligands and receptors binding, and followed by up-regulation of down-stream JAK kinases and STAT transcription factors. In addition, the STATs target genes that correlated to different biological processes, namely immune response (GO: 0006955), response to viruses (GO: 0009615), positive regulation of immune response (GO: 0050778, GO: 0002684), and JAK-related cytokine signaling (PMID: 27044867), were also shown partially elevated in Fig. 4. Such results may indicate that the splenoid lymphocytes may produce more chemokines and cytokines, and these ligands may provide positive feedback looping regulation of the proliferation and differentiation of lymphocytes in the spleen, and resulting in an overall enhancement of immune response in transgenic rainbow trout. Similar RNA-Seq results of tilapia spleen were reported by Zhu et al. [32] that the *Streptococcus iniae* challenged fish were shown significantly perturbed of expression levels of C-C/C-X-C motif chemokines, C-X-C chemokines receptors, interleukins, interferons, and MHC class I/II antigens at its acute phase after infection. Additionally, the transcriptomic analysis of spleen of Atlantic salmon conducted by Dettleff et al. [33], demonstrated that the fish altered their expression profiles of interleukins/interleukin receptors (*il10*, *il10rb*, and *il13ra2*), interferon/interferon receptors (*ifng* and *ifngr1*), in responding of viral infection of infectious salmon anemia virus (ISAV). In our transgenic rainbow trout, the Toll-like receptor signaling was also highly promoted in the spleen of transgenic fish by establishing up-regulated genes of Toll-like receptors (*tlr1*, *2*, *3*, *7* and *8*), and the important adaptor protein (*myd88*). The expression levels of the down-stream kinases (e.g. *tbk1*, *p38* and *pi3k*), transcription factors (*irf3*, *irf7* and *stat1*), and targeting genes (*cd40*, *cxcl10* and *cxcl11*) were all elevated (Additional file 3: Figure S4). The stimulated Toll-like receptor signaling may result in an enhancing chemotaxis of immune cells and increasing pro-inflammatory effects via Toll-like to NF-κB pathway. Moreover, complement components of *c1q*, *c4*, *c3ar1*, *c5ar1*, *c6* and *c7* were found up-regulated in the complement and coagulation pathway of the transgenic spleen (Additional file 3: Figure S3), and may indicate an increasing expression of complement complex in the transgenic fish. Finally, the surface integrins (*itga4* and *itgb1*) of leukocytes, essential matrix metalloproteinases (*mmp2* and *mmp9*), docking actins (*actn1* and *actb1*), and recognition receptor: *cxcr4* were increased their expression levels in leukocyte trans-endothelial migration pathway (Additional file 4: Figure S6). These findings may suggest that leukocytes might have higher recognition and adhesion abilities targeting onto epitheliums. The overall observed perturbation in the spleen by the cecropin P1 transgene product strongly support our hypothesis that enhancement of the spleen function leads to acceleration of innate/adaptive immune responses in cecropin P1 transgenic rainbow trout.

### Kidney: The major hematopoiesis relevant organ

While in the absence of bone marrow, the anterior kidney of teleost fish plays an equivalent role of hematopoiesis as bone marrow in higher vertebrates. For instance, B-lymphocytes are produced in fish kidney, as well as myeloid lineage cells (e.g. monocytes, macrophages, and granulocytes) [34, 35]. In the current study, three important functional perturbations were observed in the kidney of the transgenic rainbow trout, and the spraying RPKM ratios of DEGs to custom-made pathways for detail evaluation were performed as well. For the kidney, the gene expression profiles of (1) hematopoietic cell lineage, (2) leukocyte trans-endothelial migration, and (3) PPAR signaling pathways were obviously altered. In the hematopoietic cell lineage, the expression of many lymphocytic associated surface antigen genes were down regulated. For example, down regulation of the expression *cd2*/cd3 (pro-T cell) and *cd8* (T cell) genes was observed. In addition, the expression of *cd9*/*cd22* (pre/pro-B cell), *cd37* (immature B cell), *igm*/*igd* (B cell), *cd11b*/*cd13* (pro-monocyte) and *cd115* (monocyte) genes was down-regulated as well. By combining the finding of cDNA microarray analysis conducted by Lo et al. [12] and the results of the current study, a suppressed translational machinery is suggested and further reduced lymphocytic proliferation and differentiation may be inferred in transgenic kidney. However, enhancement of maturation and activation of lymphocytes were observed in transgenic spleen (see discussed in the spleen section), so the results from the kidney could be counter balanced by the spleen effects, and to a homeostasis of lymphocytic function been maintained in the transgenic rainbow trout. In contrast to spleen, kidney was demonstrated a suppressed leukocyte trans-endothelial migration in transgenic rainbow trout. Namely, surface integrins (*itga4*, *itgb7*), recognition antigens (*cd11a* and *cd11b*), and docking actins (*actg1* and *actn1*) were down-regulated in the transgenic kidney (Additional file 4: Figure S7). In short, the lacking of surface antigens of lymphocytes and leukocytes may be the result from decreasing production of blood cells in the early stages of hematopoietic lineage in the transgenic kidney. Our results were supported by a cross-species transcriptomic analysis conducted by Xiang et al. [36]. RNA samples from head kidney and spleen of *Vibrio harveyi* challenged Japanese sea bass (*Lateolabrax japonicus*) were sequenced, and functional perturbations of antigen presenting and processing (*mhc1/2*, *hsp70*, *hsp90*, and *calnexin*), pattern recognition (*collectin12* and *itga3*) and regulators of hematopoiesis (*gfi1* and *gfi1b*) were reported by Xiang et al. [36]. Finally, the PPAR signaling pathway was significantly promoted in the transgenic kidney, and the overall effects of enhancing PPAR signaling will be discussed in the liver section.

### Liver: The major energy efficiency relevant organ

As reviewed by Jenne et al. [37], liver was identified as one of the most important immune relevant organs in mammals [37]. Alike to mammals, fish liver also plays critical roles in their immunity [38]. Additionally, liver is responsible for many energy metabolism processes as well, including metabolic protein synthesis, degradation, and fatty acids (FA) biosynthesis [38]. According to Bransden et al. [39], salmonid immunity was highly correlated to the alteration of their FA compositions. In addition, interrupting fish energy efficiency via starvation [40] or treating fish with additional fatty acids [41] could change the innate and adaptive immune responses in Atlantic salmon. In the study by Lo et al. [12], DEGs contributing to energy metabolism pathways were observed in the transgenic liver, and they suggested that it might indirectly affect fish immunity. Here, our RNA-Seq results confirmed the findings of Lo et al. [12] that pathways of PPAR signaling and FA biosynthesis were greatly perturbed in the transgenic liver. For PPAR signaling (Additional file 3: Figure S5), FA transport protein (*slc27a1*) and binding protein (*fabp1*) were both up-regulated. Moreover, the expression levels of target genes that are responsible for lipid transportation (*pltp* and *apoa1*), FA oxidation (*acadm*), and adipocyte differentiation (*acdc* and *angptl4*) were elevated as well. An additional supportive finding was provided by other scientists in the RNA-Seq transcriptomic analysis of the liver of *Edwardsiella tarda* vaccine immunized zebrafish [42]. Yang et al. [42] reported differentially expression levels of energy metabolism associated genes such as apolipoprotein (*apoa4*), plasminogen (*plg*), fibrinogen (*fgg*) and serum amyloids (*apcs* and *saa*) in the liver of zebrafish (*Danio rerio*) at its acute phase after vaccine immunization. Very interestingly, the PPAR signaling was also perturbed in the transgenic kidney (Additional file 4: Figure S9): *slc27a1*, *fabp1*, *acdc*, *angptl4* and *acadm* were consistently up-regulated in both the liver and kidney. Although some of the DEGs involving in the PPAR signaling are down-regulated, the up-regulated DEGs suggest an overall elevation of lipid metabolism in both the transgenic liver and kidney, and, therefore, an enhancing lipid energy metabolism may be expected in the transgenic fish. As revealed by Yang et al. [42], the vaccine immunized zebrafish altered the expressions of lysozyme (*lygl1*) and lysosomal membrane protein 2 (*lamp2*) in the liver. Here, we discovered the promotion of phagosomal and lysosomal activity in the liver of cecropin P1 transgenic rainbow trout. The collectin (*colec11*) and C-lectin receptors (*cd206* and *cd209*) were stimulated as well as *mhc1*, and genes correlated to lysosomal activity were also perturbed such as *rilp*, *lamp*, and *ctsl* (Additional file 4: Figure S8). These results may indicate an increasing endoplasmic reticulum mediated phagocytosis, which play an essential role in lysosomal elimination of pathogens, in the transgenic liver.

## Conclusions

In this study, we confirmed the findings of cDNA microarray analysis conducted by Lo et al. [12] in a cross-platform approach, RNA-Seq, and more solid evidence were obtained to support our hypothesis. The cecropin P1 transgene not only directly eliminate bacterial, virial and parasitic pathogens, but also modulate the host innate and adaptive immunity in rainbow trout. Many functional perturbations found in the spleen and kidney might contribute to directly increasing fish immune responses; some others in the liver and kidney might provide indirect effects via altering the energy metabolisms. The genetic data shown in this study provides evidence, expands pool of knowledge about fish immunity, and may benefit further studies in marine species. Finally, the identified DEGs committing to achieve immunomodulation may further serve as biological markers for artificial selection and breeding of disease resistant fish species in aquaculture.

## Methods

### RNA preparation

Three immune competent tissues, namely spleen, liver and kidney, were harvested from two individual fish of two transgenic families respectively and one non-transgenic family (one year old) maintained in the Salmon Disease Laboratory at Oregon State University (OSU protocol # 4282). Prior to tissue collection, fish were euthanized by treatment with MS222 (Tricaine mesylate, Sigma-Aldrich, St. Louis, Mo) following the specification in OSU protocol #4282. For each tissue, one RNA sample was prepared from a pooled tissue of two individual fish of the control population (non-transgenic fish) and two RNA samples each was prepared from a pooled tissue of two individual fish of two independent transgenic families. Total RNA samples were prepared by the TRIzol extraction method following the protocol provided by the manufacturer (Invitrogen, Carlsbad, CA). By treating all RNA samples with RNase free DNase-I (M610A, Promega, Madison, WI), the genomic DNA contamination was removed. The quality of RNA samples were assessed by an Agilent Bioanalyzer (Agilent Technologies, Santa Clara, CA), and concentrations by a NanoDrop spectrophotometer (NanoDrop Technologies, Wilmington, DE).

### Transcriptomic sequencing and reads assembly

Illumina second generation sequencing was performed by Beijing Genomic Institute (BGI) as a commercial service. RNA samples of the non-transgenic rainbow trout were used to generate reference database. Poly(A)^+^-RNA was isolated from the total RNA by magnetic beads conjugated with oligo(dT). The poly(A)^+^-RNA was fragmented to about 200 bases, and subjected to first strand cDNA synthesis using random hexamer as primers. After addition of buffer, dNTPs, RNase H and DNA polymerase I, the second strand cDNA was synthesized. The double-strand cDNA was purified, and modified by 5′-end phosphorylation and 3′-end addition of single nucleotide adenine. After ligation to the sequencing adaptors, the suitable cDNA fragments were selected as templates for the PCR amplification, and the products were sequenced by Illumina HiSeq™ 2000. After sequencing, clean reads were collected by removal reads of adaptors, reads containing > 5% unknown nucleotides and reads with low quality bases (base quality ≦ 10). Subsequently, the transcriptomic de novo assembly was carried out with the short reads assembling program, Trinity [18] to generate unigenes. After generating reference database from non-transgenic rainbow trout, tissue specific RNA samples from two transgenic families were sequenced by the same procedure. By removing the noise, reads of adaptor, reads of unknown bases > 10%, and reads of low quality bases (base quality ≦ 5) greater than 50%, clean reads were produced and mapped to reference database with SOAPaligner/SOAP2 [21]. During the mapping, no more than two mismatches were allowed in the alignment.

### Unigene annotation and functional classification

Homology searches of the assembled unigenes were performed against public protein databases of Nr, Swiss-Prot, KEGG, and COG by blastx (the e-value < 10^− 5^). In addition, functional classification of unigenes was also conducted via Blast2GO program [43] for non-redundant GO annotation, and web gene ontology annotation plot (WEGO) program [44] for GO classification.

### Differential expression of Unigenes

The gene expression level was calculated by parameter of RPKM [18]. RPKM is defined as RPKM(Χ) = 10^6*^C/(NL*10^− 3^), where RPKM(Χ) stands for the expression of gene Χ, C equals to the number of reads that specifically aligned to gene Χ, N is the total number of reads that aligned to all genes, and L stands for the number of bases of gene Χ. After determining RPKM of unigenes, the base two logarithm of RPKM ratio of transgenic over non-transgenic group was calculated. For those unigenes with RPKM equals to zero were excluded, and the unigenes with RPKM ratio greater than 2 folds, i.e., log_2_(transgenic/non-transgenic > 1 or < − 1), were defined as differentially expressed genes (DEGs). If there was more than one unigene relevant to a gene of interest, the longest unigene was used to determine the expression level and coverage. For screening the significance of gene expression, strict algorithm was customized by BGI, as a commercial service, based on the general method of Audic et al. [45]. A false detection rate (FDR) was pre-set to a number no larger than 0.01, following the procedure of Benjanini-Hochberg [46] which was used to determine the threshold of *P*-value in multiple tests of differential expression genes.

### Reverse transcription (RT) quantitative real-time PCR (RT-qPCR)

Superscript III reverse transcriptase (18080–044, Life Technology) and oligo-(dT)_18_ were used to reverse transcribe two μg of DNase-treated RNA samples into first strand cDNA by following conditions provided by the manufacturer. The resulting cDNA products were diluted with DNase-free water into final volume of 100 μL. The qPCR assay was conducted in a 96 wells plate in the C1000 thermal cycler/CFX96 Real-Time PCR Detection System (Bio-Rad, Hercules, CA). The reaction mixture, 20 μL, contains 1 μL template, 0.5 μM gene specific primers (Additional file 5), 1x SsoFast EvaGreen Supermix (172–5201, Bio-Rad), and 0.01 μM fluorescein (170–8780, Bio-Rad). The amplification program consisted of initial denaturing for 2 min at 98 °C, followed by 40 cycles of 5 s at 98 °C and 30 s at 59 °C for annealing and synthesis. For quality control of the amplified products, a melting curve, 65 °C to 95 °C with 0.5 °C increments every 5 s, was performed after each amplification. Subsequently, the threshold cycle (Ct) values were collected by CFX manager (Bio-Rad) and analyzed by standard normalizer of 2^-ΔΔCt^ to determine the differential expression (transgenic vs non-transgenic). For the assay of each gene, the efficiencies of reverse transcription and PCR were determined to be within the limit of reproducibility.

### Bioinformatics analysis

In the enrichment analysis by GeneCodis [20], both pre-defined DEGs of each tissue from the transgenic fish and the total genes of the non-transgenic fish library, serving as a reference gene list, were inputted to inquire GO: Biological Process, GOSlim Process and KEGG pathways. The default statistical parameters (minimum number of genes: 3, statistical test: hypergeometric and chi-square; *p*-value correction = false detection rate: FDR) were applied for both singular and modular enrichment analyses.

For the software versions, databases and parameters, utilized in this study, were summarized in Additional file 6.

## Additional files

Additional file 1: Supplemental figures for pie charts of qualities of clean reads and results of sequencing saturation analyses to show quality of aligning, and supplemental table to show overall mapping results (to reference genes) per each sample. (PDF 206 kb)

Additional file 2: Singular enrichment analyses among three tissues, grouped by both GOSlim biological process and KEGG terms. (PDF 121 kb)

Additional file 3: Supplement figures for pathways generated from custom-made visualization tool. (PDF 743 kb)

Additional file 4: Supplement figures from KEGG database and sprayed by the DEGs expression profiles. (PDF 240 kb)

Additional file 5: Primers for RT-qPCR analysis. (XLSX 11 kb)

Additional file 6: Summarizing of software versions, databases and parameters for Bioinformatics analyses. (XLSX 9 kb)

## Abbreviations

AMP: Antimicrobial peptide
BGI: Beijing Genomic Institute
BLAST: Nucleotide Basic Local Alignment Search Tool
COG: Clusters of orthologous
*cox2*: Cyclooxygenase-2
DEG: Differentially expressed gene
ECM: Extracellular matrix
FA: Fatty acid
FDR: False detection rate
GO: Gene Ontology
GO:BP: Gene Ontology Biological Process
IHNV: Infectious hematopoietic necrosis virus
IHNV: Infectious Hematopoietic Necrosis Virus
*il1β*: Interleukin-1β
ISAV: Infectious salmon anemia virus
JAK: Janus kinase
KEGG: Kyoto Encyclopedia of Genes and Genomes
MHC: Major histocompatibility complex
MMC: Melanomacrophages center
NGS: Next generation sequencing
ORA: Over-representation analysis
PPAR: Peroxisome proliferator-activated receptor
qRT-PCR: Quantitative reverse transcription poly chain reaction
RNA-Seq: RNA sequencing
RPKM: Reads per kilobase of transcript per million mapped reads
SOAP: Short oligonucleotide analysis package
STAT: Signal transducer and activator of transcription
TLR: Toll-like receptor
WEGO: Web gene ontology annotation plot
