# Supplementary material for: RNA-Seq analysis of differentially expressed genes relevant to innate and adaptive immunity in cecropin P1 transgenic rainbow trout (Oncorhynchus mykiss)
Source: BMC Genomics. 2018 Oct 19;19:760. doi: 10.1186/s12864-018-5141-8 (PMC6195682; doi:10.1186/s12864-018-5141-8)
Supplement: Supplementary file 1 — Supplemental figures for pie charts of qualities of clean reads and results of sequencing saturation analyses to show quality of aligning, and supplemental table to show overall mapping results (to reference genes) per each sample. (PDF 206 kb) [file 12864_2018_5141_MOESM1_ESM.pdf]

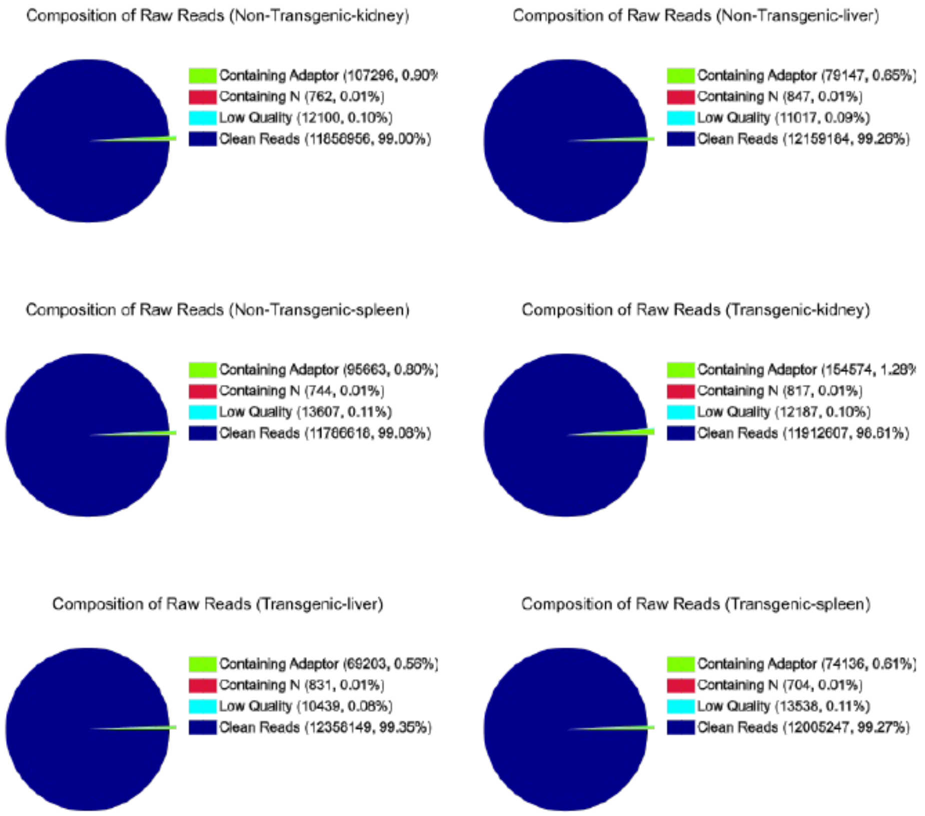

Supplemental Figure 1 Tissue specific quality assessment of reads. Pie charts contained composition of raw reads in each tissue in group of transgenic and non-transgenic fish.

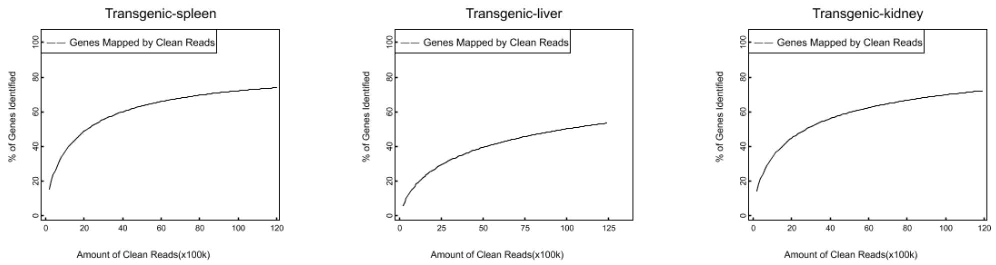

Supplemental Figure 2 Tissue specific quality of aligning: results of sequence saturation analysis. Each graph indicates the amount of clean reads of transgenic fish mapped to reference genome that reaches saturation no later than 7500k reads.

Supplemental Table 1 Summary of mapping to reference genes per each sample.

Sample ID	Total Reads	Total BasePairs	Total Mapped Reads	Perfect Match	<=2bp Mismatch	Unique Match	Multi-position Match	Total Unmapped Reads
Non-Transgenic-kidney	11,858,956(100.00%)	581,088,844(100.00%)	9,430,911(79.53%)	7,460,761(62.91%)	1,970,150(16.61%)	7,344,928(61.94%)	2,085,983(17.59%)	2,428,045(20.47%)
Non-Transgenic-liver	12,159,184(100.00%)	595,800,016(100.00%)	10,262,784(84.40%)	8,336,861(68.56%)	1,925,923(15.84%)	8,161,361(67.12%)	2,101,423(17.28%)	1,896,400(15.60%)
Non-Transgenic-spleen	11,786,618(100.00%)	577,544,282(100.00%)	9,108,348(77.28%)	7,057,019(59.87%)	2,051,329(17.40%)	7,034,848(59.69%)	2,073,500(17.59%)	2,678,270(22.72%)
Transgenic-kidney	11,912,607(100.00%)	583,717,743(100.00%)	9,435,608(79.21%)	7,179,370(60.27%)	2,256,238(18.94%)	7,565,341(63.51%)	1,870,267(15.70%)	2,476,999(20.79%)
Transgenic-liver	12,358,149(100.00%)	605,549,301(100.00%)	10,311,807(83.44%)	7,937,101(64.23%)	2,374,706(19.22%)	8,023,228(64.92%)	2,288,579(18.52%)	2,046,342(16.56%)
Transgenic-spleen	12,005,247(100.00%)	588,257,103(100.00%)	9,151,918(76.23%)	6,979,032(58.13%)	2,172,886(18.10%)	6,922,491(57.66%)	2,229,427(18.57%)	2,853,329(23.77%)
