# Supplementary material for: RNA-Seq analysis of differentially expressed genes relevant to innate and adaptive immunity in cecropin P1 transgenic rainbow trout (Oncorhynchus mykiss)
Source: BMC Genomics. 2018 Oct 19;19:760. doi: 10.1186/s12864-018-5141-8 (PMC6195682; doi:10.1186/s12864-018-5141-8)
Supplement: Supplementary file 2 — Singular enrichment analyses among three tissues, grouped by both GOSlim biological process and KEGG terms. (PDF 121 kb) [file 12864_2018_5141_MOESM2_ESM.pdf]

Spleen

Singular enrichment of GOSlim process

Genes	NGR	TNGR	NG	TNG	Hyp	Hyp*	Chi	Chi*	Annotations
29 genes	198	5674	29	384	5.36E-05	0.0017704	4.98E-05	0.00164185	GO:0007155: cell adhesion (BP)
4 genes	11	5674	4	384	0.00465134	0.0767471	0.00121573	0.0200595	GO:0007005: mitochondrion organization (BP)

Spleen

Singular enrichment of KEGG

Not available

NGR = Number of annotated genes in the reference list;
TNGR = Total number of genes in the reference list;
NG = Number of annotated genes in the input list;
TNG = Total number of genes in the input list;
Hyp = Hypergeometric pValue; Hyp* = Corrected hypergeometric pValue
Chi = Chi square pValue; Chi* = Corrected chi square pValue

Liver

Singular enrichment of GOSlim process

Genes	NGR	TNGR	NG	TNG	Hyp	Hyp*	Chi	Chi*	Annotations
2 genes	14	5674	2	99	0.0239265	0.143559	0.000876076	0.0105129	GO:0006259: DNA metabolic process (BP)
3 genes	35	5674	3	99	0.0225216	0.180173	0.00323959	0.0259167	GO:0007267: cell-cell signaling (BP)
4 genes	46	5674	4	99	0.00812107	0.194906	0.000585669	0.0140561	GO:0006461: protein complex assembly (BP)
5 genes	90	5674	5	99	0.0199786	0.239744	0.00722919	0.0433751	GO:0006629: lipid metabolic process (BP)

Liver

Singular enrichment of KEGG

Genes	NGR	TNGR	NG	TNG	Hyp	Hyp*	Chi	Chi*	Annotations
4 genes	25	5674	4	99	0.000832454	0.0674288	5.10E-07	1.03E-05	(KEGG) 00500: Starch and sucrose metabolism
2 genes	6	5674	2	99	0.00431887	0.11661	3.84E-07	1.04E-05	(KEGG) 00591: Linoleic acid metabolism
2 genes	5	5674	2	99	0.00291232	0.117949	4.34E-08	3.51E-06	(KEGG) 01040: Biosynthesis of unsaturated fatty acids
2 genes	9	5674	2	99	0.0100171	0.135231	2.54E-05	0.000257671	(KEGG) 00053: Ascorbate and aldarate metabolism
2 genes	9	5674	2	99	0.0100171	0.135231	2.54E-05	0.000257671	(KEGG) 00100: Steroid biosynthesis
3 genes	23	5674	3	99	0.00708287	0.143428	0.000110175	0.000991575	(KEGG) 03320: PPAR signaling pathway
2 genes	11	5674	2	99	0.0149603	0.173112	0.000144303	0.00116885	(KEGG) 00040: Pentose and glucuronate interconversions
1 genes	1	5674	1	99	0.017448	0.176661	1.44E-07	5.81E-06	(KEGG) 00400: Phenylalanine, tyrosine and tryptophan biosynthesis
2 genes	18	5674	2	99	0.0384567	0.183235	0.00425045	0.0181203	(KEGG) 00590: Arachidonic acid metabolism
2 genes	18	5674	2	99	0.0384567	0.183235	0.00425045	0.0181203	(KEGG) 04975: Fat digestion and absorption
2 genes	13	5674	2	99	0.0207415	0.186673	0.000519395	0.00350592	(KEGG) 05323: Rheumatoid arthritis
1 genes	2	5674	1	99	0.0345946	0.186811	2.45E-05	0.000330648	(KEGG) 00360: Phenylalanine metabolism
1 genes	2	5674	1	99	0.0345946	0.186811	2.45E-05	0.000330648	(KEGG) 00130: Ubiquinone and other terpenoid-quinone biosynthesis
2 genes	16	5674	2	99	0.030848	0.192207	0.00210192	0.010015	(KEGG) 00140: Steroid hormone biosynthesis
2 genes	16	5674	2	99	0.030848	0.192207	0.00210192	0.010015	(KEGG) 00830: Retinol metabolism
3 genes	45	5674	3	99	0.0431273	0.194073	0.0150859	0.0469984	(KEGG) 04060: Cytokine-cytokine receptor interaction
2 genes	20	5674	2	99	0.0466978	0.19908	0.00758636	0.0292617	(KEGG) 04115: p53 signaling pathway
2 genes	15	5674	2	99	0.0272978	0.201011	0.00139149	0.00867002	(KEGG) 00983: Drug metabolism - other enzymes
4 genes	65	5674	4	99	0.0262361	0.212513	0.00859709	0.0316529	(KEGG) 04062: Chemokine signaling pathway

NGR = Number of annotated genes in the reference list;
TNGR = Total number of genes in the reference list;
NG = Number of annotated genes in the input list;
TNG = Total number of genes in the input list;
Hyp = Hypergeometric pValue; Hyp* = Corrected hypergeometric pValue
Chi = Chi square pValue; Chi* = Corrected chi square pValue

Kidney

Singular enrichment of GOSlim process

Genes	NGR	TNGR	NG	TNG	Hyp	Hyp*	Chi	Chi*	Annotations
1 genes	1	5674	1	243	0.0428269	0.235548	0.0010714	0.0176781	GO:0021700: developmental maturation (BP)
1 genes	1	5674	1	243	0.0428269	0.235548	0.0010714	0.0176781	GO:0002376: immune system process (BP)

Kidney

Singular enrichment of KEGG

Genes	NGR	TNGR	NG	TNG	Hyp	Hyp*	Chi	Chi*	Annotations
7 genes	23	5674	7	243	3.30E-05	0.005078	1.04E-07	1.60E-05	(KEGG) 03320: PPAR signaling pathway
5 genes	16	5674	5	243	0.0004096	0.012614	5.17E-06	0.000199	(KEGG) 00140: Steroid hormone biosynthesis
4 genes	9	5674	4	243	0.0003488	0.013431	1.24E-06	6.35E-05	(KEGG) 00020: Citrate cycle (TCA cycle)
4 genes	9	5674	4	243	0.0003488	0.013431	1.24E-06	6.35E-05	(KEGG) 04672: Intestinal immune network for IgA production
10 genes	73	5674	10	243	0.0009918	0.021821	0.000241	0.0041243	(KEGG) 04810: Regulation of actin cytoskeleton
4 genes	11	5674	4	243	0.0008535	0.021907	1.04E-05	0.0002672	(KEGG) 00620: Pyruvate metabolism
6 genes	23	5674	6	243	0.0003166	0.024377	6.45E-06	0.0001987	(KEGG) 04640: Hematopoietic cell lineage
5 genes	23	5674	5	243	0.0024671	0.042215	0.0002377	0.0045758	(KEGG) 05410: Hypertrophic cardiomyopathy (HCM)
3 genes	7	5674	3	243	0.002389	0.045988	3.63E-05	0.0007995	(KEGG) 00120: Primary bile acid biosynthesis
5 genes	25	5674	5	243	0.0036323	0.055938	0.0005101	0.0078559	(KEGG) 05412: Arrhythmogenic right ventricular cardiomyopathy (ARVC)
5 genes	26	5674	5	243	0.0043429	0.0608	0.0007199	0.0100783	(KEGG) 05414: Dilated cardiomyopathy
6 genes	39	5674	6	243	0.0057645	0.073977	0.0017433	0.0223722	(KEGG) 04512: ECM-receptor interaction
6 genes	40	5674	6	243	0.0065445	0.077527	0.0021679	0.0256807	(KEGG) 04514: Cell adhesion molecules (CAMs)
6 genes	45	5674	6	243	0.0116277	0.119378	0.0056444	0.0482905	(KEGG) 04060: Cytokine-cytokine receptor interaction
4 genes	21	5674	4	243	0.0110453	0.121498	0.0026742	0.0294158	(KEGG) 05120: Epithelial cell signaling in Helicobacter pylori infection
3 genes	13	5674	3	243	0.0161296	0.137998	0.0031212	0.0300414	(KEGG) 04610: Complement and coagulation cascades
3 genes	13	5674	3	243	0.0161296	0.137998	0.0031212	0.0300414	(KEGG) 05323: Rheumatoid arthritis
4 genes	23	5674	4	243	0.0152845	0.147114	0.0049511	0.0448508	(KEGG) 04145: Phagosome
2 genes	7	5674	2	243	0.0332651	0.213451	0.0061318	0.0472145	(KEGG) 00260: Glycine, serine and threonine metabolism
2 genes	7	5674	2	243	0.0332651	0.213451	0.0061318	0.0472145	(KEGG) 00380: Tryptophan metabolism

NGR = Number of annotated genes in the reference list;
TNGR = Total number of genes in the reference list;
NG = Number of annotated genes in the input list;
TNG = Total number of genes in the input list;
Hyp = Hypergeometric pValue; Hyp* = Corrected hypergeometric pValue
Chi = Chi square pValue; Chi* = Corrected chi square pValue
