# Supplementary material for: RNA-Seq analysis of differentially expressed genes relevant to innate and adaptive immunity in cecropin P1 transgenic rainbow trout (Oncorhynchus mykiss)
Source: BMC Genomics. 2018 Oct 19;19:760. doi: 10.1186/s12864-018-5141-8 (PMC6195682; doi:10.1186/s12864-018-5141-8)
Supplement: Supplementary file 3 — Supplement figures for pathways generated from custom-made visualization tool. (PDF 743 kb) [file 12864_2018_5141_MOESM3_ESM.pdf]

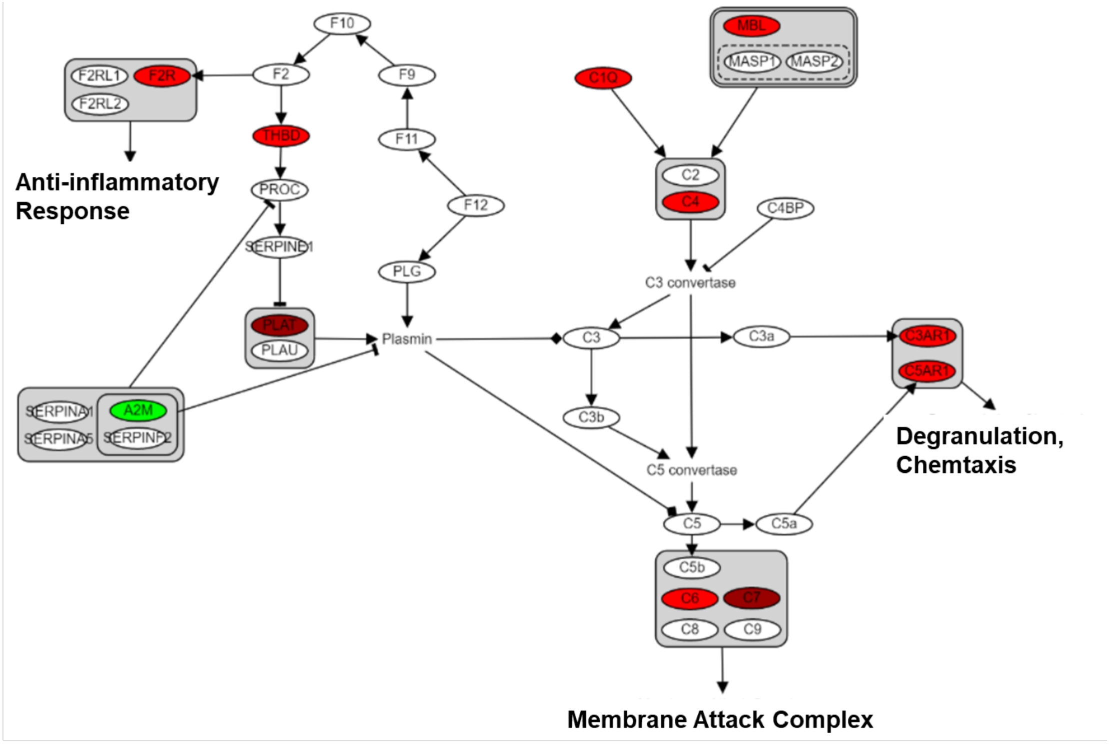

Supplement Figure 3 Pathway analysis of complement cascade and coagulation pathway in transgenic spleen. The heat map indicates: red = up-regulation, green = down-regulation. Threshold set = RPKM ratio greater than two folds. Eclipse = regular gene; dash line bundle = set of OR; singular rectangular bundle = set of AND; doubling rectangular bundle = set of AND out of subset of OR. The pathway was customized and sprayed expression data from KO04610 (complement and coagulation cascades) from KEGG database.

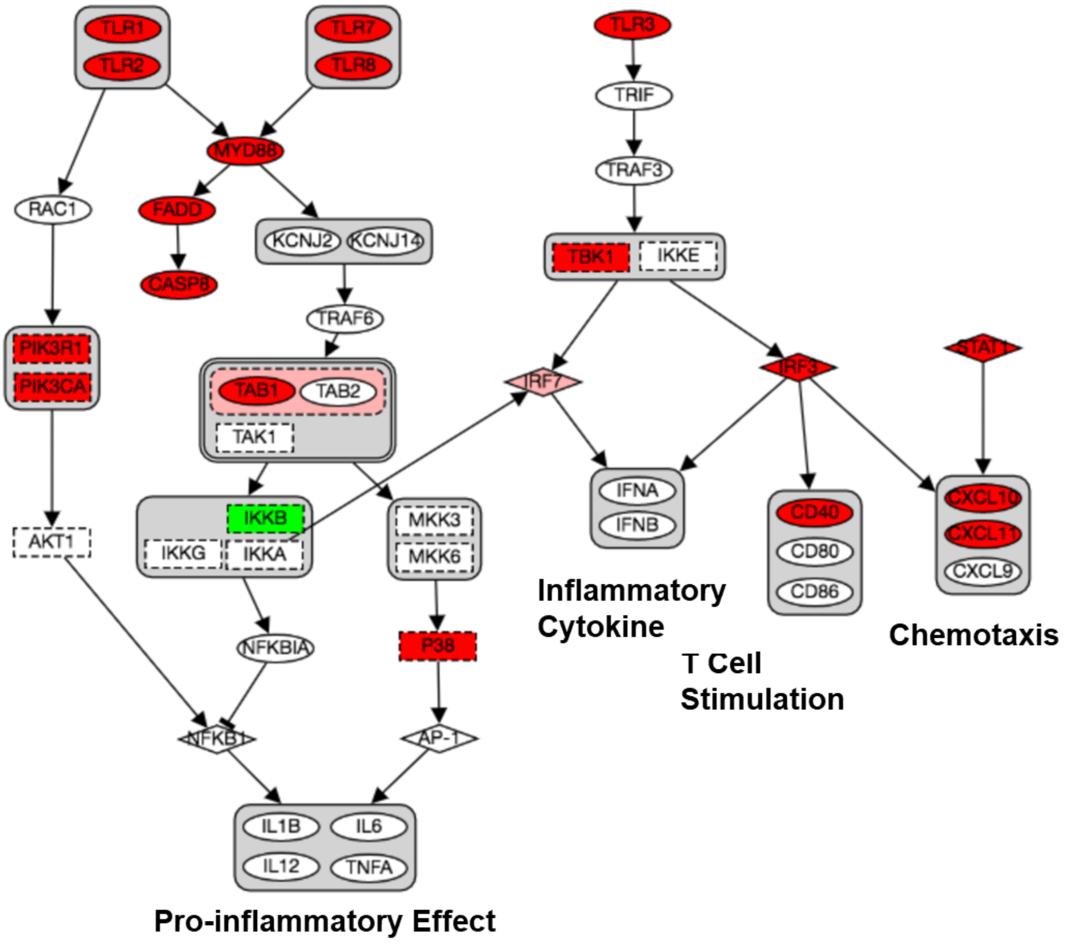

Supplement Figure 4 Pathway analysis of Toll-like receptor pathway in transgenic spleen. The heat map indicates: red = up-regulation, green = down-regulation. Threshold set = RPKM ratio greater than two folds. Eclipse = regular genes; dash rectangular = kinases; diamond = transcription factors; dash line bundle = set of OR; singular rectangular bundle = set of AND; doubling rectangular bundle = set of AND out of subset of OR. The pathway was customized and sprayed expression data from KO04620 (Toll-like receptor signaling pathway) from KEGG database.

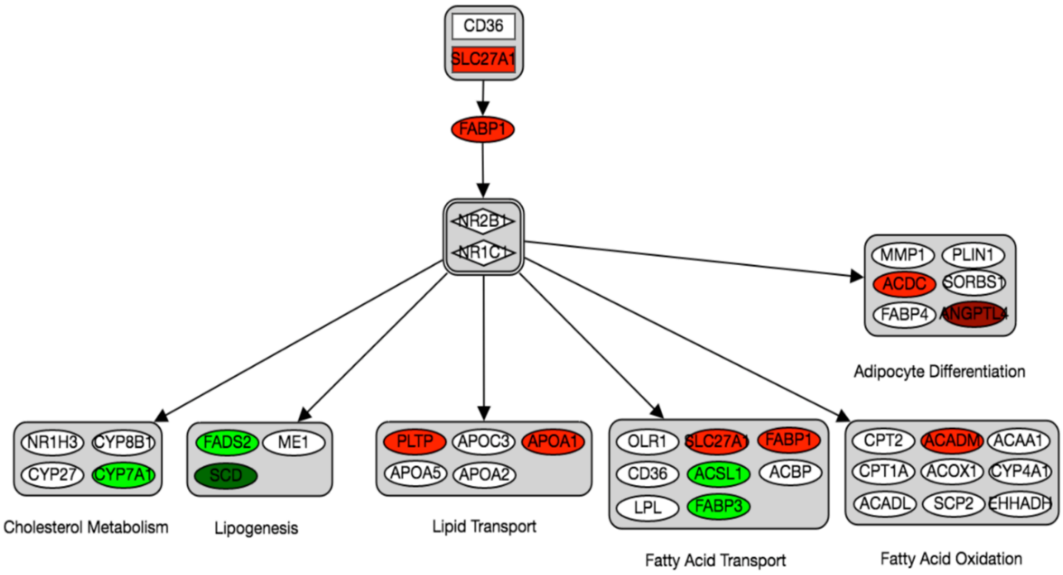

Supplement Figure 5 Pathway analysis of PPAR signaling pathway in transgenic liver. The heat map indicates: red = up-regulation, green = down-regulation. Threshold set = RPKM ratio greater than two folds. Eclipse = regular genes; diamond = transcription factors; singular rectangular bundle = set of AND; doubling rectangular bundle = either AND/OR. The pathway was customized and sprayed expression data from KO03320 (PPAR signaling pathway) from KEGG database.
