# Supplementary material for: RNA-Seq analysis of differentially expressed genes relevant to innate and adaptive immunity in cecropin P1 transgenic rainbow trout (Oncorhynchus mykiss)
Source: BMC Genomics. 2018 Oct 19;19:760. doi: 10.1186/s12864-018-5141-8 (PMC6195682; doi:10.1186/s12864-018-5141-8)
Supplement: Supplementary file 4 — Supplement figures from KEGG database and sprayed by the DEGs expression profiles. (PDF 240 kb) [file 12864_2018_5141_MOESM4_ESM.pdf]

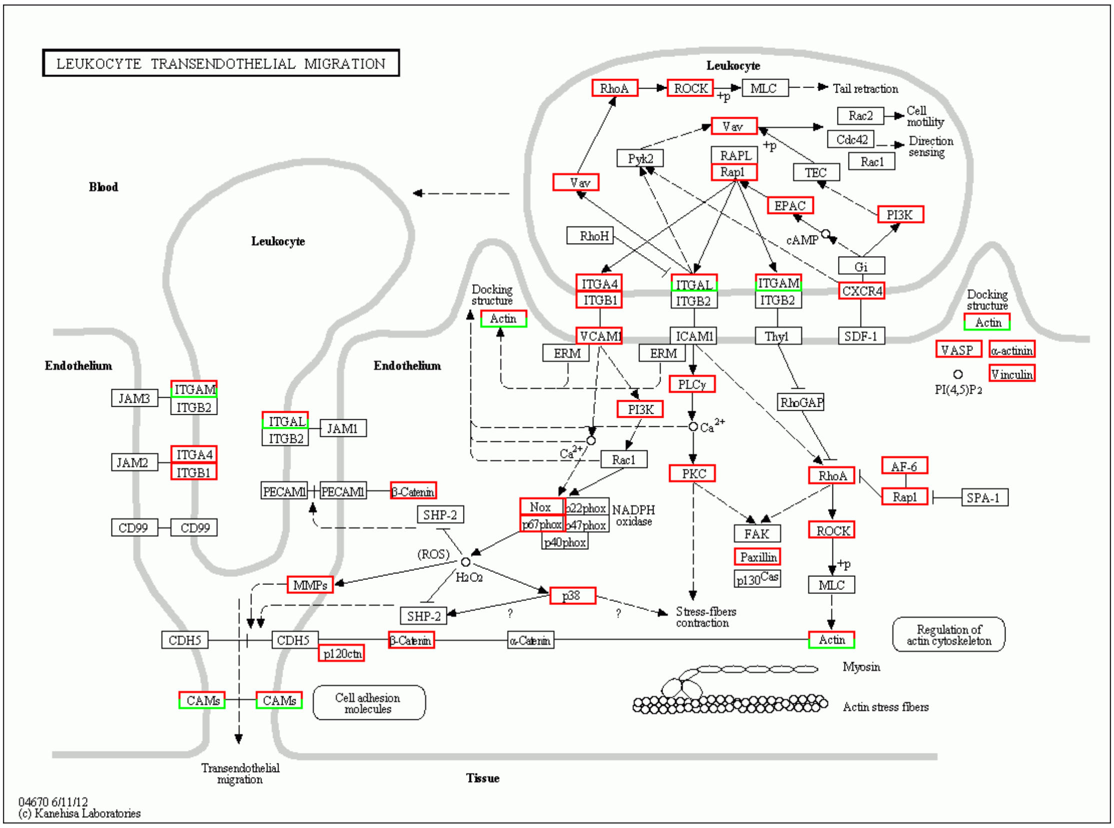

Supplement Figure 6 KEGG Pathway of leukocyte trans-endothelial migration in transgenic spleen. Red coated rectangular = up-regulation; Green coated rectangular = down-regulation; Double coated rectangular = isomers with differentially expressed levels. Threshold set = RPKM ratio greater than two folds. The pathway was directly inquired from KEGG database (KO04670) and sprayed spleen expression data.

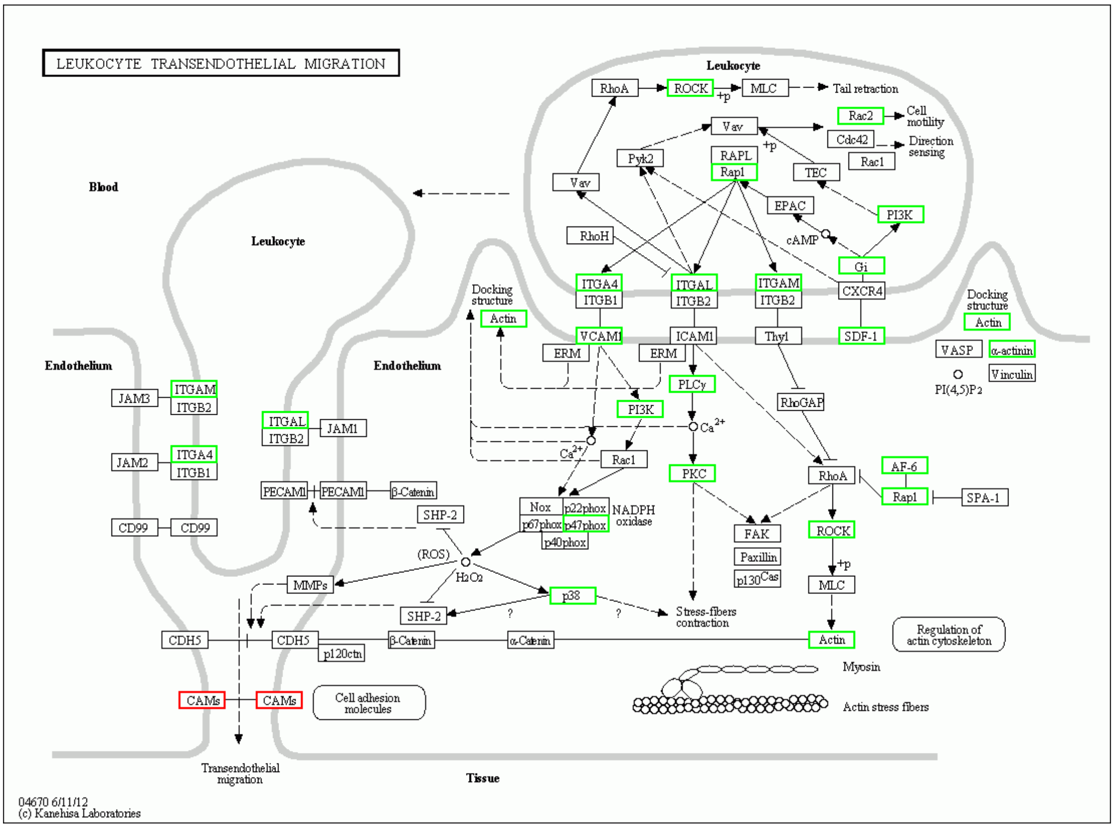

Supplement Figure 7 KEGG Pathway of leukocyte trans-endothelial migration in transgenic kidney. Red coated rectangular = up-regulation; Green coated rectangular = down-regulation; Double coated rectangular = isomers with differentially expressed levels. Threshold set = RPKM ratio greater than two folds. The pathway was directly inquired from KEGG database (KO04670) and sprayed kidney expression data.

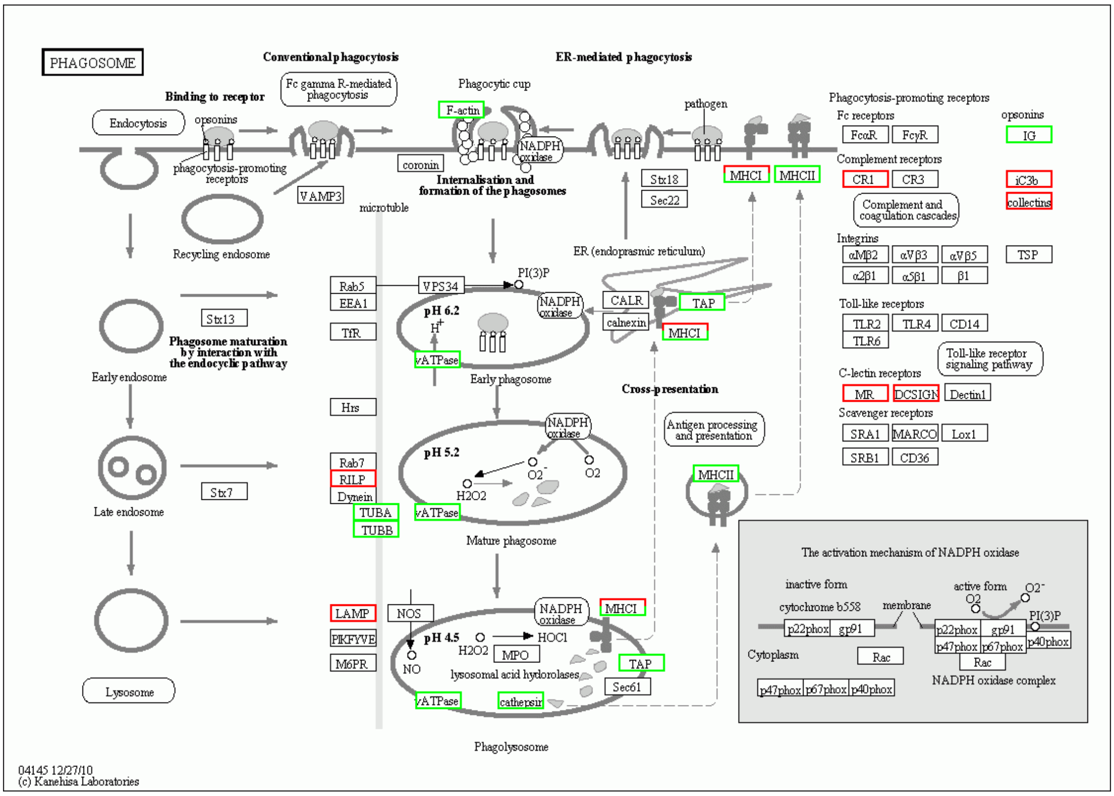

Supplement Figure 8 KEGG Pathway of Phagosome in transgenic liver. Red coated rectangular = up-regulation; Green coated rectangular = down-regulation; Double coated rectangular = isomers with differentially expressed levels. Threshold set = RPKM ratio greater than two folds. The pathway was directly inquired from KEGG database (KO04145) and sprayed liver expression data.

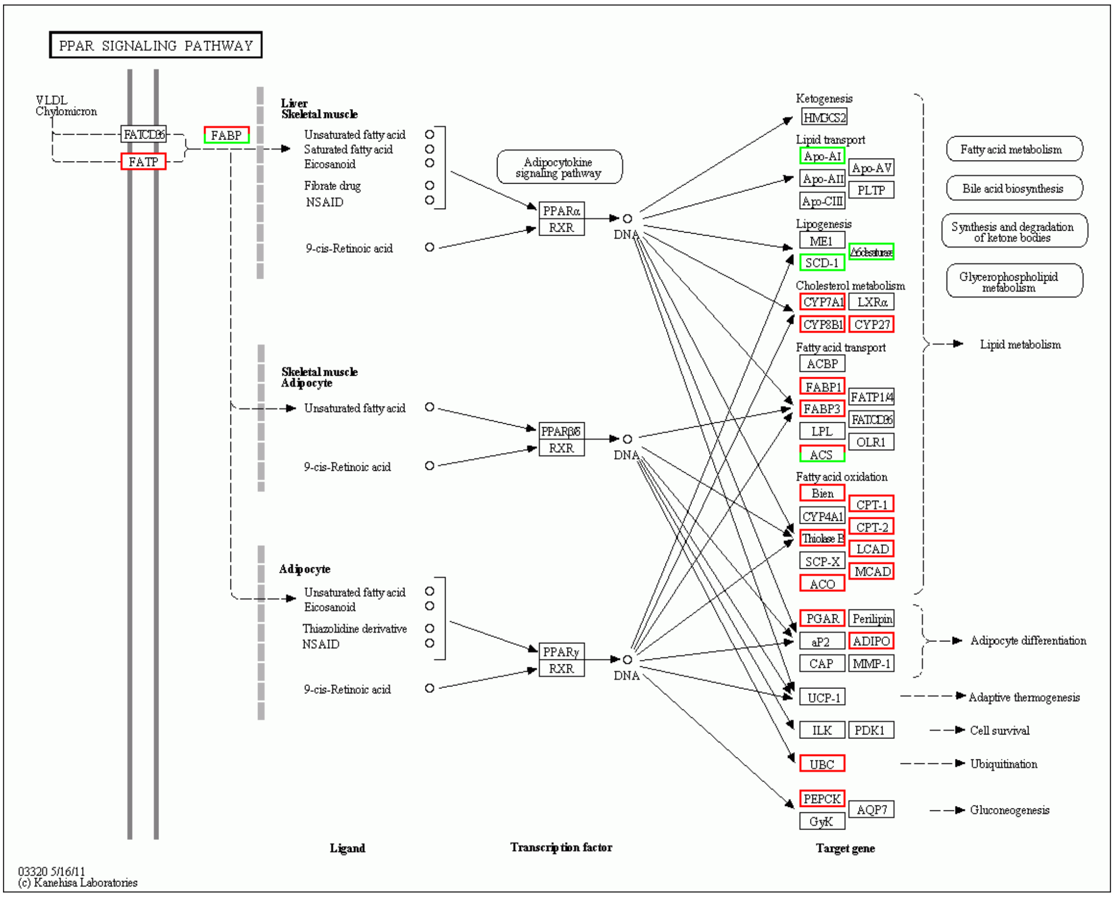

Supplement Figure 9 KEGG Pathway of PPAR in transgenic Kidney. Red coated rectangular = up-regulation; Green coated rectangular = down-regulation; Double coated rectangular = isomers with differentially expressed levels. Threshold set = RPKM ratio greater than two folds. The pathway was directly inquired from KEGG database (KO03320) and sprayed kidney expression data.
